# Clarification of the taxonomic status of *Acanthochitonadiscrepans* (Brown, 1827) with new data for the North-East Atlantic *Acanthochitona* (Polyplacophora, Acanthochitonidae)

**DOI:** 10.3897/BDJ.11.e109554

**Published:** 2023-11-28

**Authors:** Katarzyna Vončina, Nina Therese Mikkelsen, Christine Morrow, Rory Ang, Julia D. Sigwart

**Affiliations:** 1 Department of Marine Zoology, Senckenberg Research Institute and Natural History Museum Frankfurt, Frankfurt am Main, Germany Department of Marine Zoology, Senckenberg Research Institute and Natural History Museum Frankfurt Frankfurt am Main Germany; 2 Department of Natural History, University of Bergen, Bergen, Norway Department of Natural History, University of Bergen Bergen Norway; 3 Queen's University Marine Laboratory, Queen’s University, Portaferry, Ireland Queen's University Marine Laboratory, Queen’s University Portaferry Ireland; 4 University of Exeter, Exeter, United Kingdom University of Exeter Exeter United Kingdom

**Keywords:** *Acanthochitona*, geographic ranges, integrative taxonomy, North-East Atlantic chitons

## Abstract

**Background:**

The genus *Acanthochitona* can be easily distinguished from other chitons by having eighteen tufts of bristles on the dorsal side of the densely spiculose girdle. In the North-East Atlantic, five species of this genus have been recognised so far: *A.crinita* (Pennant, 1777), *A.discrepans* (Brown, 1827), *A.fascicularis* (Linnaeus, 1767), *A.oblonga* Leloup, 1968 and *A.pilosa* Schmidt-Petersen, Schwabe et Haszprunar, 2015. The nomenclature of *A.crinita*, *A.discrepans* and *A.fascicularis* was confused for a very long time until Kaas (1985) designated type specimens for them and provided a brief key. However, his work lacked detailed descriptions of the three species and some authors doubted that *A.discrepans* constitutes a separate species. Subsequently, the taxonomic status of *A.discrepans* has remained unclear.

**New information:**

Here, we implemented an integrative approach which combined morphology and molecular evidence to show that *Acanthochitonadiscrepans* is, indeed, a valid species and we present re-descriptions for *A.crinita*, *A.discrepans* and *A.fascicularis*.

## Introduction

The class Polyplacophora, also known as chitons, comprise one of the eight extant classes of molluscs. These exclusively marine animals can be found worldwide, from the intertidal zone to the deep sea (Eernisse 2004, Sigwart 2009, Schwabe 2010). There are around 1000 extant species known; however, the true species richness is likely higher as marine biodiversity tends to be underestimated (Bouchet 2023). Chitons can be recognised by their eight articulating, usually overlapping dorsal valves, surrounded by a girdle, which bears different kinds of ornamentation (Stebbins and Eernisse 2000, Schwabe 2010). The genus *Acanthochitona* can be easily distinguished from other genera by 18 prominent tufts of bristles on the dorsal side of the girdle.

In the North-East Atlantic, five species of *Acanthochitona* have been recognised so far: *A.crinita* (Pennant, 1777), *A.discrepans* (Brown, 1827), *A.fascicularis* (Linnaeus, 1767), *A.oblonga* Leloup, 1981 and *A.pilosa* Schmidt-Petersen, Schwabe et Haszprunar, 2015 (Kaas 1985, Dell’Angelo and Smriglio 2001, Bonfitto et al. 2011, Schmidt-Petersen et al. 2015). Since their original descriptions, the taxonomy of *A.crinita*, *A.discrepans* and *A.fascicularis* has been very confused. Kaas (1985) attempted to resolve this issue by designating type specimens for each species; he also provided an identification key (for the detailed history and bibliography of the nomenclatural confusion, see Kaas (1985)). Shortly thereafter, an influential regional key stated that there was no reason to consider *A.discrepans* as separate from *A.crinita* (Jones and Baxter 1987) and, since then, the taxonomic status of *A.discrepans* has remained unclear.

In the current study, we used morphological and molecular data of *Acanthochitona* from the North-East Atlantic using freshly-collected specimens and museum material. An integrative approach showed that *A.discrepans* is a valid species; additionally, we included re-descriptions for *A.crinita*, *A.discrepans* and *A.fascicularis*.

## Materials and methods

Specimens for SEM analysis were dissected and then the valves, girdle and radula were transferred on to a glass cube with bleach and left until the rest of the tissue had dissolved. Prepared parts were gold-sputtered and examined in CamScan "CS 24" from Cambridge Instruments available at the Senckenberg Research Institute in Frankfurt. All figures were assembled in Adobe Photoshop CS6.

For DNA barcoding, a small piece of tissue from the chiton foot was sampled. DNA from the specimens ZSM20040263 and ZSM20150336 was extracted using QIAamp DNA Micro Kit (QIAGEN). Specimens SMF 373026–36, ZMBN140293–6, ZMBN146755 were sent to BOLD for the extraction and sequencing (The Barcode of Life Data System, https://www.boldsystems.org/). Specimens ZMBN140331–33, ZMBN138238, SMF 363941–3, SMF 360506 and SMF 373024–5 were extracted using the Qiagen DNeasy kit and amplified using TaKaRa Taq HS. For extraction procedures, the manufacturers’ protocols were followed. The cytochrome oxidase subunit I (COI primers LCO1490 and HCO2198; Folmer et al. (1994)) was amplified using repliQa HiFi ToughMix from ThermoFisher, following the PCR programme for COI in Bonfitto et al. (2011). The sequences were manually inspected in Geneious Prime v.2023.1. Additionally, fifteen COI sequences from GenBank, labelled as *Acanthochitonacrinita*, one COI sequence labelled as *A.fascicularis* and one sequence of *Craspedochitonlaqueatus* (used as an outgroup), were downloaded from GenBank and aligned with the new sequences from this study using default settings of MAFFT7 (Katoh et al. 2002, Katoh and Toh 2008) under the Q-INS-I strategy. Aligned sequences were trimmed to the length of 618 bp. Uncorrected pairwise distances were calculated using MEGA11 (Kumar et al. 2016). A COI phylogeny was calculated using the GTR+G, GTR+I and F81+I models for subsequent partitions corresponding with different codon positions, suggested by PartitionFinder version 2.1.1 (Lanfear et al. 2017) using Bayesian Information Criterion (BIC) and greedy algorithm (Lanfear et al. 2012). Bayesian Inference (BI) marginal posterior probabilities were calculated using MrBayes v.3.2 (Ronquist and Huelsenbeck 2003). Random starting trees were used and the analysis was run for three million generations, sampling the Markov chain every 1000 generations.

The new sequences are publicly available in The Barcode of Life Data System (BOLD, https://www.boldsystems.org) and GenBank (https://www.ncbi.nlm.nih.gov/genbank/); see "Materials" sections of each species for their accession numbers (as "associatedSequences"). The list of DNA sequences downloaded from GenBank, the alignment and genetic distances are available as Suppl. materials 1, 2, 3.

## Taxon treatments

### Acanthochitona crinita

(Pennant, 1777)

7D51C117-F2F1-523B-87E3-6688A511319C

https://www.marinespecies.org/aphia.php?p=taxdetails&id=138675

Chiton crinitus : Pennant, 1777 - Pennant 1777: 71, pl. 36, Figs. 1, A1.
non Chitoncrinitus: Sowerby II, 1840a - Sowerby II 1840a: figs 88-93; 1840b - Sowerby II 1840b: 2.
Chiton onyx : Spengler, 1797 - Spengler 1797: 95; Kaas, 1981 - Kaas 1981: 220, fig. 6.
non *Chitononyx*: Morch, 1870 - Mörch 1870: 113.
*Chitonfascicularis*: Brown, 1827 - Brown 1827: pl. 35 fig. 8 (not fig. 5); 1844 -Brown 1844: 65, pl. 21 fig. 8 (not fig. 5); Sowerby II, 1840a -Sowerby II 1840a: figs. 87, 87a; 1840a - Sowerby II 1840b: 1; Sowerby II, 1859 - Sowerby II 1859, pl. 10, fig. 5; Forbes & Hanley, 1849 -Forbes and Hanley 1849: 393, pl. 59 fig. 5; Hanley, 1855 -Hanley 1855: 15; Jeffreys, 1865 - Jeffreys 1865: 211; 1869 - Jeffreys 1869: 197, pl. 55 fig. 3; et mult auct.
non *Chitonfascicularis*: Linnaeus, 1767 - Linnaeus 1767.
Chiton fascicularis var. minor : Philippi, 1836 - Philippi 1836: 108.
Acanthochaetes vulgaris : Leach, 1852 - Leach 1852: 229, pl. 10 fig. 8.
Chiton fascicularis var. attenuata : Jeffreys, 1865 - Jeffreys 1865: 212.
Acanthochites aeneus : di Monterosato, 1878a - di Monterosato 1878: 147.
non *Acanthochitesaeneus*: Risso, 1826 - Risso 1826.
Acanthochites (ton) *adansoni*: de Rochebrune, 1881a - de Rochebrune 1881a: 44; 1881b - de Rochebrune 1881b: 116; 1881c - de Rochebrune 1881c: 238, pl. 17 figs. 9a-b; Pilsbry, 1893 - Pilsbry 1893: 13, pl. 8 figs. 33-34; Thiele, 1909 - Thiele 1909: 43, pl. 5 figs. 69-73; Bergenhayn, 1931 - Bergenhayn 1931: 28, pl. 3 fig. 81; Leloup, 1968 - Leloup 1968: 61, figs. 3-7, 11, 14.
Acanthochites (tori) bouvieri: de Rochebrune, 1881a - de Rochebrune 1881a: 45; 1881b - de Rochebrune 1881b: 117; 1881c - de Rochebrune 1881c: 239, pls. 17 figs. 10a, b; Pilsbry, 1893 - Pilsbry 1893: 13, pl. 3 figs. 65-66; Thiele, 1909 - Thiele 1909: 42; Leloup, 1968 - Leloup 1968: 62, figs. 4-7, 14.
Anisochiton (Acanthochites) fascicularis var. violacea : Pallary, 1902 - Pallary 1902: 29.
Acanthochites fascicularis vars *var. lutescens*, *cinnabrina et fusca*: Dautzenberg & Durouchoux, 1906 - Dautzenberg and Durouchoux 1906: 15.
Acanthochitona crinitus : Winckworth, 1926 - Winckworth 1926: 15; 1932: 218.
Acanthochiton fascicularis : Leloup, 1936 - Leloup 1936: 3, fig. 3; 1968 - Leloup 1968: 60, figs. 1-6, 8-11, 13-14 (bibliography); et mult. auct.
non *Chitonfascicularis*: Linnaeus, 1767 - Linnaeus 1767.
Acanthochiton gracilis : Leloup, 1968 - Leloup 1968: 74 (ex parte).
*non Chitongracilis* Jeffreys, 1859 - Jeffreys 1859.
Acanthochitona crinita : Kaas, 1985 - Kaas 1985: 588, Figs. 7-50.
**Type material.** Neotype: RSMNH 1978.052.02601, Royal Scottish Museum of Natural History, Edinburgh, Scotland. Designated by Kaas (1985) (material not seen).
**Type locality.** Scotland, sea near Aberdeen (Pennant 1777); Scotland, Monach Islands, North Uist, 57°31.5'N, 07°38.5'W (Kaas 1985).

#### Materials

- **Type status:** Other material. **Occurrence:** individualID: SMF 373026; individualCount: 1; lifeStage: adult; otherCatalogNumbers: BOLD:AAY5203, NIB_CCM_0542; associatedSequences: BOLD:CCMMO049-21, GenBank: OR526579; occurrenceID: F0AAA8D3-4C51-551B-95F3-0C69454D56D4; **Taxon:** scientificNameID: Acanthochitonacrinita; **Location:** country: England; locality: Falmouth; verbatimCoordinates: 50°08'38"N 5°03'46"W; **Event:** eventDate: 08/10/2021; eventRemarks: Rory Ang and Jessie Dermody leg.; **Record Level:** collectionCode: Malakologie- SMF; basisOfRecord: PreservedSpecimen
- **Type status:** Other material. **Occurrence:** individualID: SMF 363941-3; individualCount: 1; lifeStage: adult; associatedSequences: GenBank: OR145408; occurrenceID: 602565CB-D2C4-5C78-85DB-C21D0702B889; **Taxon:** scientificNameID: Acanthochitonacrinita; **Location:** country: Portugal; stateProvince: Azores; county: São Miguel; locality: Ponta Delgada, Rosto do Cão; verbatimCoordinates: 37°44.567'N 25°38.167'W; **Event:** eventDate: 29/07/2013; eventRemarks: Julia Sigwart, Laura Sumner-Rooney & Nicholas Carey leg.; **Record Level:** collectionCode: Malakologie- SMF; basisOfRecord: PreservedSpecimen
- **Type status:** Other material. **Occurrence:** individualID: SMF 363942-1; individualCount: 1; lifeStage: adult; associatedSequences: GenBank: OR145404; occurrenceID: F1F6B54D-AC5D-5102-97A0-B0BF71B35A28; **Taxon:** scientificNameID: Acanthochitonacrinita; **Location:** country: Portugal; stateProvince: Azores; county: São Miguel; locality: Capelas, São Vicente; verbatimCoordinates: 37°50.4'N 25°40.8'W; **Event:** eventDate: 28/07/2013; eventRemarks: Julia Sigwart, Laura Sumner-Rooney & Nicholas Carey leg.; **Record Level:** collectionCode: Malakologie- SMF; basisOfRecord: PreservedSpecimen
- **Type status:** Other material. **Occurrence:** individualID: SMF 363942-2; individualCount: 1; lifeStage: adult; associatedSequences: GenBank: OR145405; occurrenceID: FDAF4402-13F8-5CCB-A272-A493FA0D2FD3; **Taxon:** scientificNameID: Acanthochitonacrinita; **Location:** country: Portugal; stateProvince: Azores; county: São Miguel; locality: Capelas, São Vicente; verbatimCoordinates: 37°50.4'N 25°40.8'W; **Event:** eventDate: 28/07/2013; eventRemarks: Julia Sigwart, Laura Sumner-Rooney & Nicholas Carey leg.; **Record Level:** collectionCode: Malakologie- SMF; basisOfRecord: PreservedSpecimen
- **Type status:** Other material. **Occurrence:** individualID: SMF 363941-1; individualCount: 1; lifeStage: adult; associatedSequences: GenBank: OR145406; occurrenceID: 4863948B-0D53-5E02-B0C1-5AEFBCACC547; **Taxon:** scientificNameID: Acanthochitonacrinita; **Location:** country: Portugal; stateProvince: Azores; county: São Miguel; locality: Ponta Delgada, Rosto do Cão; verbatimCoordinates: 37°44.567'N 25°38.167'W; **Event:** eventDate: 29/07/2013; eventRemarks: Julia Sigwart, Laura Sumner-Rooney & Nicholas Carey leg.; **Record Level:** collectionCode: Malakologie- SMF; basisOfRecord: PreservedSpecimen
- **Type status:** Other material. **Occurrence:** individualID: SMF 363943; individualCount: 1; lifeStage: adult; associatedSequences: GenBank: OR145403; occurrenceID: 4C33B9E9-B6CD-502D-80B1-E3E99435E35A; **Taxon:** scientificNameID: Acanthochitonacrinita; **Location:** country: Portugal, Ilha de São Miguel, Ponta Delgada, Rosto do Cão; stateProvince: Azores; county: São Miguel; locality: Ponta Delgada, Rosto do Cão; verbatimCoordinates: 37°44.567'N 25°38.167'W; **Event:** eventDate: 29/07/2013; eventRemarks: Julia Sigwart, Laura Sumner-Rooney & Nicholas Carey leg.; **Record Level:** collectionCode: Malakologie- SMF; basisOfRecord: PreservedSpecimen
- **Type status:** Other material. **Occurrence:** individualID: SMF 363941-2; individualCount: 1; lifeStage: adult; associatedSequences: GenBank: OR145407; occurrenceID: B71A3E64-B60E-5247-8F90-D598F186FD1B; **Taxon:** scientificNameID: Acanthochitonacrinita; **Location:** country: Portugal; stateProvince: Azores; county: São Miguel; locality: Ponta Delgada, Rosto do Cão; verbatimCoordinates: 37°44.567'N 25°38.167'W; **Event:** eventDate: 29/07/2013; eventRemarks: Julia Sigwart, Laura Sumner-Rooney & Nicholas Carey leg.; **Record Level:** collectionCode: Malakologie- SMF; basisOfRecord: PreservedSpecimen
- **Type status:** Other material. **Occurrence:** individualID: ZSM20150336; individualCount: 1; lifeStage: adult; associatedSequences: GenBank: OR145411; occurrenceID: F70C7D1C-8366-5231-AEAC-95AB7AF1AC1E; **Taxon:** scientificNameID: Acanthochitonacrinita; **Location:** country: France; stateProvince: Brittany, Finistère; locality: Roscoff; verbatimCoordinates: 48°43'40''N 3°59'21''W; **Event:** eventDate: 10/06/2013; eventRemarks: Gerhard Haszprunar leg.; **Record Level:** institutionCode: SNSB-ZSM; collectionCode: ZSM-Mol; basisOfRecord: PreservedSpecimen

#### Description

**Figure 1.**
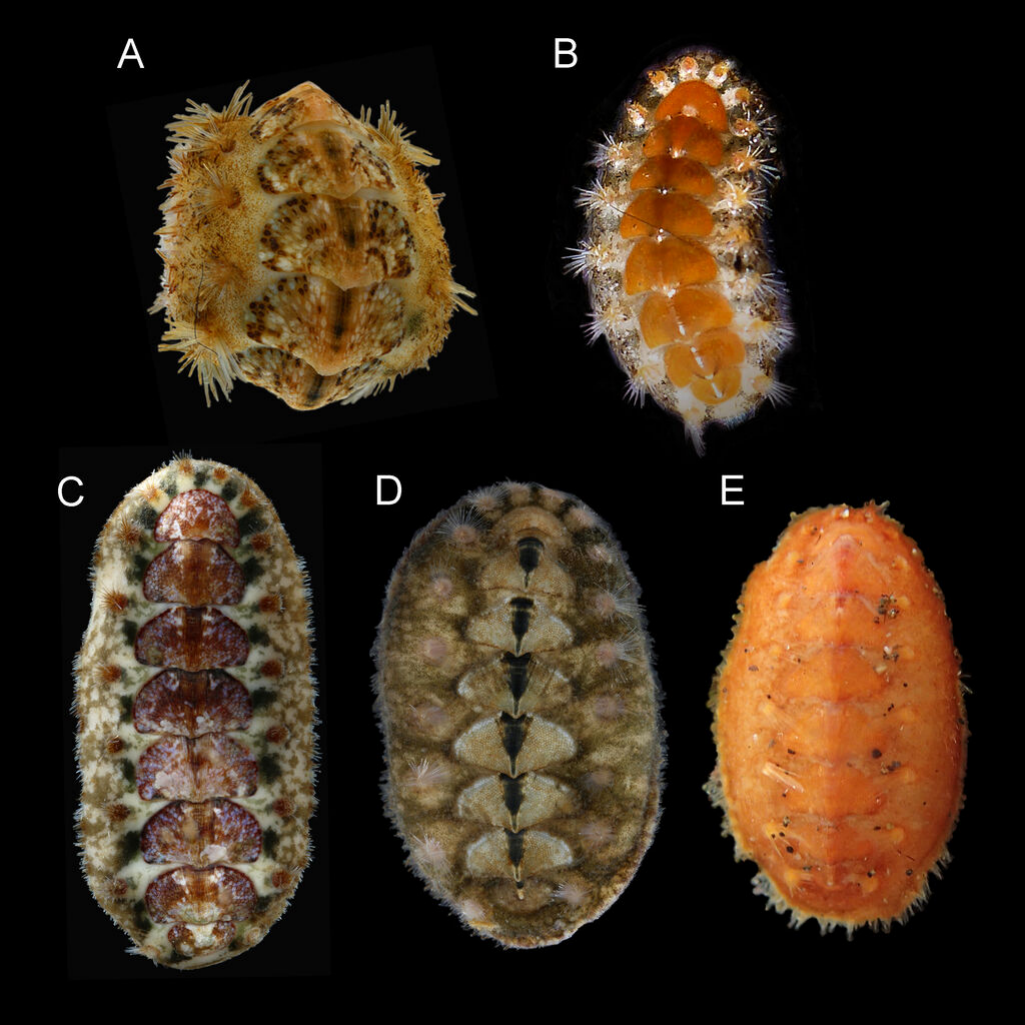
Images of the adult specimens of *Acanthochitona*, dorsal view. **A** *A.crinita* from England, BL not measured; **B** *A.crinita* from the Azores, BL not measured; **C** *A.discrepans* from Norway, BL 17 mm; **D** *A.fascicularis* from Ireland, BL 23 mm; **E** *A.fascicularis* from the Azores, BL 13 mm.

(See Jones and Baxter (1987), Dell’Angelo and Smriglio (2001), Bonfitto et al. (2011), Mitov (2015), Schmidt-Petersen et al. (2015) for additional descriptions that pertain to *A.crinita* s.s.). Small to medium-sized (in examined material, BL: 8–18 mm, BW: 4–10 mm, BL/BW ratio: 2), outline oval, moderately elevated, semi-carinated, slightly protruding apices; girdle wide, spiculose. Tegmentum colouration not uniform, mottled and very variable (Fig. 1A–B).

**Figure 2.**
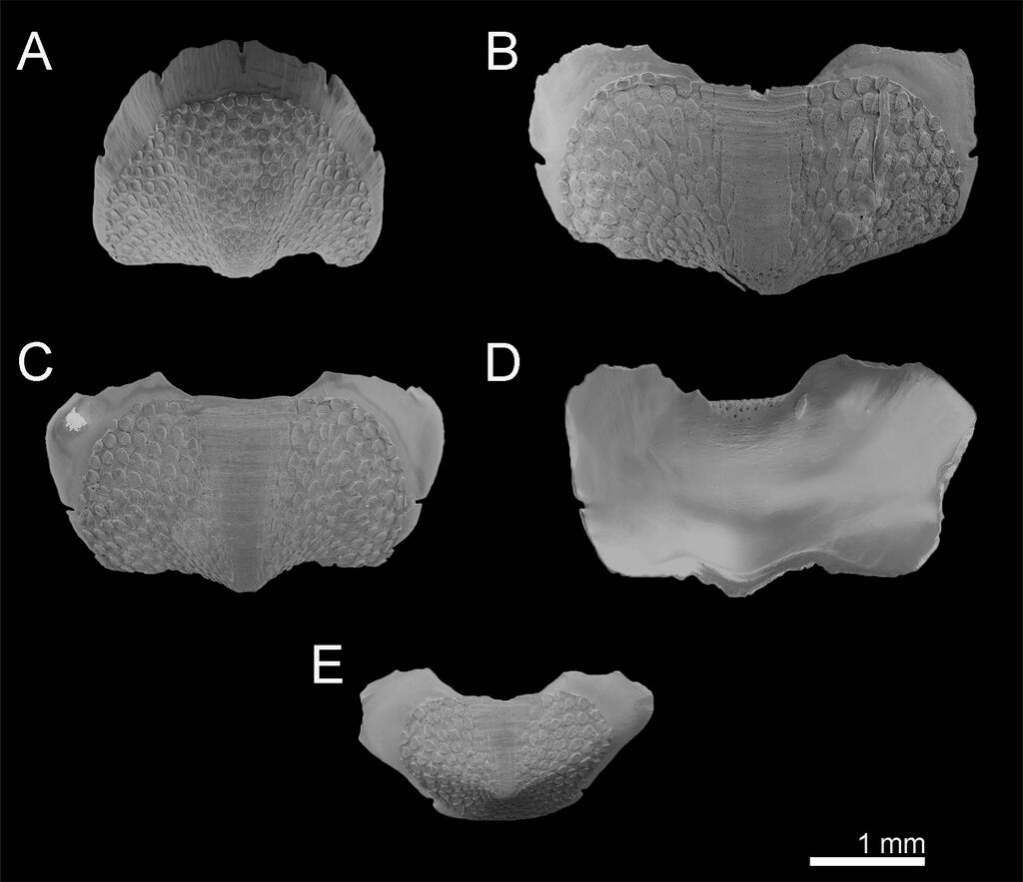
*Acanthochitonacrinita* from the Azores (A, C, E) and England (B, D). **A** Valve I, dorsal view; **B** Valve VI, dorsal view; **C** Valve VII, dorsal view; **D** Valve VII, ventral view; **E** Valve VIII, dorsal view.

Head valve slightly wider than long, almost semicircular, five rays barely raised, apex clearly visible (Fig. 2A). Intermediate valves ellipsoidal, wide, anterior margin slightly convex at both sides of straight or little concave jugum; hardly raised striated jugal area (Fig. 1A–B, Fig. 2B–C). Tail valve roughly semicircular with central, slightly elevated mucro (Fig. 2E).

**Figure 3.**
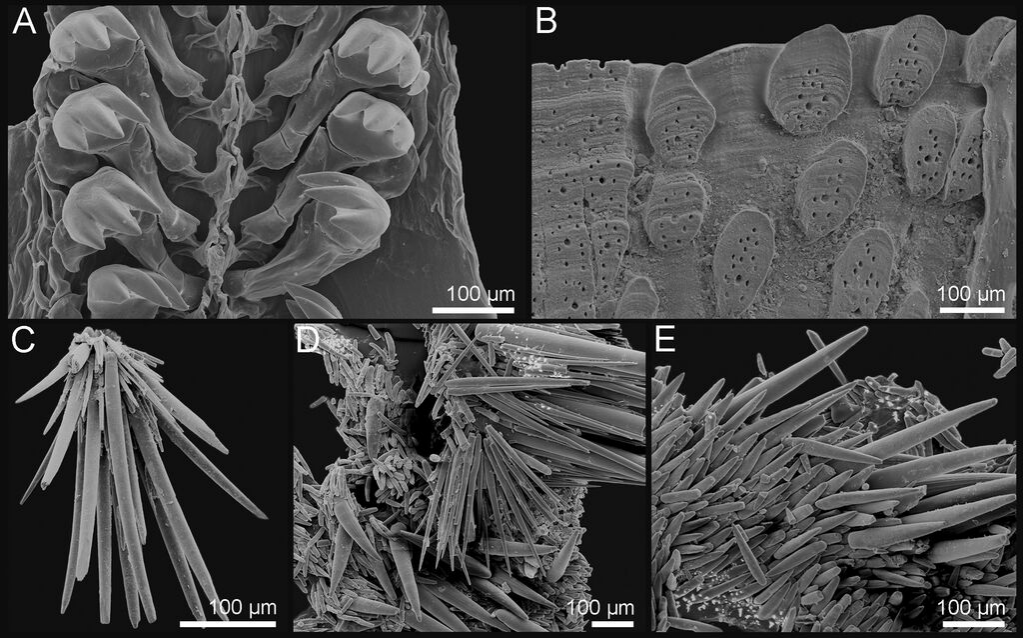
*Acanthochitonacrinita* from the Azores (A, D, E) and England (B, C). **A** Radula; **B** Valve VI, details of the tegmentum in the lateropleural area; **C** One of the 18 tufts of spicules on the girdle; **D, E** Tuft, dorsal and marginal spicules.

**Figure 4.**
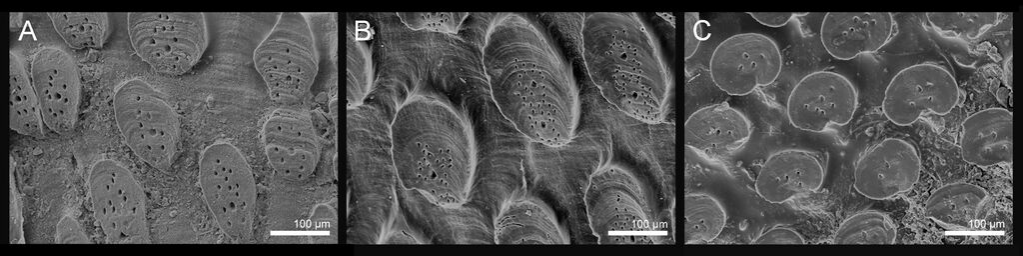
Morphology of the granules of tegmentum with the macro- and microaesthetes. **A** *Acanthochitonacrinita*; **B** *A.discrepans*; **C** *A.fascicularis*.

Tegmentum covered with densely packed to widely separated (density from 23 to 38 granules per 1 mm^2^, mean = 31, n = 8 specimens) granules of variable shape, from oval to more or less elongate drop-shaped; granules raised, flat-topped, with one macroaesthete subcentral surrounded by 8–16 posteromedially located microaesthetes (Fig. 1A, Fig. 2A–C, E, Fig. 3B and Fig. 4A).

Articulamentum thick, apophyses large, broadly rectangular, rounded at anterolateral margins, separated by a wide sinus; insertion plates wide, continuous with apophyses, slits moderately deep (slit formula: 5/1/2) (Fig. 2).

Girdle wide, fleshy, usually brighter than tegmentum, deeply encroaching sutural areas. Highly variable colour of the tegmentum and perinotum; usually from creamy-white to olive-green and brown, with different degrees of blotches in which white, yellow and brown variously combined; in some specimens, colours can be very bright, for example, bright yellow and orange. (Fig. 1A–B). Perinotum covered with two kinds of spicules: evenly distributed, short, somewhat bent and distally striated spicules (up to 100 x 15 µm) and longer thick spicules (up to 260 x 40 µm), striated (some of them smooth), usually slightly curved, randomly interspersed amongst them (Fig. 3D). Perinotum ornamentation highly variable, observed variability in examined specimens: dorsal ornamentation just as described above or only short and densely arranged spicules or densely arranged, short but very thin, irregularly-curved spicules, with long and thick spicules interspersed amongst them. Sutural tufts with around 20–46 thick, smooth needles (up to 340 x 20 µm) intermingled with much shorter and thinner needles (Fig. 3C). Marginal fringe with long and finely-ribbed spicules (some spicules may have deep grooves); hyponotum beset with slightly flattened, distally finely striated spicules, similar to the short dorsal spicules, up to 110 x 18 µm (Fig. 3E).

Radula central tooth elongated, almost straight at the top and slightly keeled near base; first lateral tooth wing-shaped and smaller than the central; second (major) lateral elongated, with accessory plate tricuspidate, outer denticle slightly shorter than the others; cusps pointed (Fig. 3A).

Gills merobranchial, 13–15 ctenidia on each side.

#### Diagnosis

*Acanthochitonacrinita* is well known for its high variability of morphological features. However, it can be distinguished from other NE Atlantic relatives on the basis of the below-mentioned morphological characters. *A.crinita* can be distinguished from *A.discrepans* (Brown, 1827), based on the number, size and arrangement of aesthetes on tegmental granules (one macroaesthete subcentral surrounded by larger, posteromedially-located 8–16 microaesthetes in *A.crinita* vs. 1–2 macroaesthetes: a single macroaesthete located in posterior third, second, if present, located centrally, surrounded by very small, posteriomedially located 26–40 microaesthetes in *A.discrepans*), dorsal spicules (longer, thicker spicules up to 100 x 15 µm in *A.crinita* vs. smaller, thinner spicules up to 68 x 7.5 µm in *A.discrepans*), tuft needles (longer spicules up to 340 x 20 µm, surrounded by a smaller number of very short and thin needles in *A.crinita* vs. longer spicules up to 1000 µm x 60 µm, surrounded by a large number of somewhat shorter and much thinner needles in *A.discrepans*). *A.crinita* differs from *A.fascicularis* (Linnaeus, 1767) by the shape of the intermediate valves (ellipsoidal in *A.crinita* vs. triangular in *A.fascicularis*), jugal area (hardly raised, not sharply separated from the latero-pleural areas in *A.crinita* vs. raised, sharply separated from the latero-pleural areas in *A.fascicularis*), shape of dorsal granules (moderately to widely apart, oval to elongated oval in *A.crinita* vs. small, round, densely packed granules with the incision in the middle in *A.fascicularis*), density of tegmental granules (23–38 granules per 1 mm^2^, mean = 31 in *A.crinita* vs. 42–70 granules per 1 mm^2^, mean = 52 in *A.fascicularis*), number of microaesthetes (8–16 microaesthetes in *A.crinita* vs. 0–6 microaesthetes in *A.fascicularis*), number of bristles in the sutural tufts (20–46 in *A.crinita* vs. 55-120 in *A.fascicularis*). It differs from *A.oblonga* Leloup, 1981 by the shape of dorsal granules (moderately apart, oval to elongated oval in *A.crinita* vs. widely apart and very much elongated in *A.oblonga*), number and arrangement of microaesthetes (8–16 microaesthetes located posteromedially in *A.crinita* vs. 6–9 microaesthetes, located mainly in the central area in *A.oblonga*). It can be easily distinguished from *A.pilosa* Schmidt-Petersen, Schwabe et Haszprunar, 2015 by the shape of the IV valve (valve with the hind margin concave at both sides of the pronounced apex in *A.crinita* vs. valve with triangular posterior margin with no apex in *A.pilosa*), density of tegmental granules (moderately to widely apart in *A.crinita* vs. densely packed in *A.pilosa*), size of microaesthetes on the tegmental granules (bigger microaesthetes in *A.crinita* vs. smaller, ca. ½ of the diameter, in *A.pilosa*), large dorsal spicules (not always present, sparse, curved spicules of size up to 260 x 40 µm in *A.crinita* vs. always present, dense and straight spicules of size up to 320 × 60-62 µm in *A.pilosa*).

#### Distribution

*Acanthochitonacrinita* is a widely distributed species in North-East Atlantic. The species inhabits the seas from of north of Scotland (*locus typicus*), south of England (molecular data and SEM photos from this study), north of France and Spain, west coast of Portugal (molecular data from GenBank), the Azores (molecular data and SEM photos from this study) and, most likely, the Mediterranean Sea (literature data).

### Acanthochitona discrepans

(Brown, 1827)

67A9570B-CD67-53EF-8E45-B795D6051492

https://www.marinespecies.org/aphia.php?p=taxdetails&id=138676

Chiton discrepans : Brown, 1827 - Brown 1827, pl. 35 fig. 20; 1844 - Brown 1844: 65, p. 21 fig. 20.
non *Chitondiscrepans*: Sowerby II, 1840a: 2 [in synonymy of *C.crinitus* (non Pennant)] et mult. auct.
Chiton fascicularis : Brown, 1827 - Brown 1827 (ex parte): pl. 35 fig. 5; 1844 - Brown 1844: 65 (ex parte), pl. 21 fig. 5.
non *Chitonfascicularis*: Linnaeus, 1767 - Linnaeus 1767.
Chiton gracilis : Jeffreys, 1859 - Jeffreys 1859: 106, pl. 3 figs 9a-c; Sowerby II, 1859 - Sowerby II 1859: pl. 10 fig. 6; Winckworth, 1926 - Winckworth 1926: 15, pl. 1, fig. 1, lb-d.
Chiton fascicularis var. gracilis : Jeffreys, 1865 - Jeffreys 1865: 212; Dean, 1926 - Dean 1926: 21; Pilsbry, 1893 - Pilsbry 1893: 11, pl. 4 fig. 83.
Acanthochitona discrepans : Winckworth, 1926 - Winckworth 1926: 15, pl. 1 fig. 2; Dean, 1926 - Dean 1926: 21; Winckworth, 1932 - Winckworth 1932: 218.
Chiton gracile : Warén, 1980 - Warén 1980: 13.
Acanthochtona crinita : Kaas, 1985 - Kaas 1985: 591, Figs. 28-38.
Acanthochitona discrepans : Kaas, 1985 - Kaas 1985: 598, Figs. 59-75.
**Type material.** Lectotype: TENBM 1983.4588/1, Tenby Museum, Tenby, Pembroke, Wales. Designated by Kaas (1985) (material not seen).
**Type locality.** Wales, Pembroke, Tenby (Pennant 1777, Kaas 1985).

#### Materials

- **Type status:** Other material. **Occurrence:** individualID: SMF 373031; individualCount: 1; lifeStage: adult; otherCatalogNumbers: BOLD:ADZ1926, NIB_CCM_0087; associatedSequences: BOLD:NIB087-21, GenBank: OR526580; occurrenceID: F0EE7AE3-9B89-526A-835A-EFD87BE7FF37; **Taxon:** scientificNameID: Acanthochitonadiscrepans; **Location:** country: Northern Ireland; county: Down; locality: Strangford Lough; verbatimCoordinates: 54°23'38"N 5°34'40"W; **Event:** eventDate: 14/02/2021; eventRemarks: Christine Morrow, Bernard Picton & Julia Sigwart leg.; **Record Level:** collectionCode: Malakologie- SMF; basisOfRecord: PreservedSpecimen
- **Type status:** Other material. **Occurrence:** individualID: SMF 373033; individualCount: 1; lifeStage: adult; otherCatalogNumbers: BOLD:ADZ1926, NIB_CCM_0343; associatedSequences: BOLD:CCMMO021-21, GenBank: OR526581; occurrenceID: 2F5A0B87-0B67-5DD9-9AB4-D7E2D35306BF; **Taxon:** scientificNameID: Acanthochitonadiscrepans; **Location:** country: Northern Ireland; county: Down; locality: Strangford Lough; verbatimCoordinates: 54°23'38"N 5°34'40"W; **Event:** eventDate: 09/04/2021; eventRemarks: Christine Morrow leg.; **Record Level:** collectionCode: Malakologie- SMF; basisOfRecord: PreservedSpecimen
- **Type status:** Other material. **Occurrence:** individualID: SMF 373032; individualCount: 1; lifeStage: adult; otherCatalogNumbers: BOLD:ADZ1926, NIB_CCM_0334; associatedSequences: BOLD:CCMMO018-21, GenBank: OR526582; occurrenceID: F07D922B-2D26-5E53-B753-CD66EB7CC5B1; **Taxon:** scientificNameID: Acanthochitonadiscrepans; **Location:** country: Northern Ireland; county: Down; locality: Strangford Lough; verbatimCoordinates: 54°23'38"N 5°34'40"W; **Event:** eventDate: 09/04/2021; eventRemarks: Christine Morrow leg.; **Record Level:** collectionCode: Malakologie- SMF; basisOfRecord: PreservedSpecimen
- **Type status:** Other material. **Occurrence:** individualID: SMF 373036; individualCount: 1; lifeStage: adult; otherCatalogNumbers: BOLD:ADZ1926, NIB_CCM_0476; associatedSequences: BOLD:CCMMO041-21, GenBank: OR526585; occurrenceID: 5E9A5A5A-5676-5772-BC76-253093721D8A; **Taxon:** scientificNameID: Acanthochitonadiscrepans; **Location:** country: Northern Ireland; county: Down; locality: Strangford Lough; verbatimCoordinates: 54°29'34"N 5°39'03"W; **Event:** eventDate: 25/05/2021; eventRemarks: Christine Morrow leg.; **Record Level:** collectionCode: Malakologie- SMF; basisOfRecord: PreservedSpecimen
- **Type status:** Other material. **Occurrence:** individualID: SMF 373024; individualCount: 1; lifeStage: adult; associatedSequences: GenBank: OR145402; occurrenceID: 1FFACC15-2880-5B42-A013-76F661C8B18E; **Taxon:** scientificNameID: Acanthochitonadiscrepans; **Location:** country: Northern Ireland; county: Down; locality: Strangford Lough; verbatimCoordinates: 54°29'23"N 5°32'15"W; **Event:** eventDate: 19/09/2019; eventRemarks: Julia Sigwart leg.; **Record Level:** collectionCode: Malakologie- SMF; basisOfRecord: PreservedSpecimen
- **Type status:** Other material. **Occurrence:** individualID: ZMBN140332; individualCount: 1; lifeStage: adult; otherCatalogNumbers: BOLD:ADZ1926; associatedSequences: BOLD:NORCH109-23, GenBank: OR526587; occurrenceID: CA9290A1-1E55-5CF3-9F3E-1A8011E24634; **Taxon:** scientificNameID: Acanthochitonadiscrepans; **Location:** country: Norway; stateProvince: Trondelag; locality: Hopavågen; verbatimCoordinates: 63°35'35"N 9°32'0"E; **Event:** eventDate: 14/10/2020; eventRemarks: Nina T. Mikkelsen leg.; **Record Level:** institutionCode: ZMBN; basisOfRecord: PreservedSpecimen
- **Type status:** Other material. **Occurrence:** individualID: ZMBN140293; individualCount: 1; lifeStage: adult; otherCatalogNumbers: BOLD:ADZ1926; associatedSequences: BOLD:NORCH005-21, GenBank: OR526590; occurrenceID: 487EB93E-0FDE-5C67-8A8E-A955D0A4495C; **Taxon:** scientificNameID: Acanthochitonadiscrepans; **Location:** country: Norway; stateProvince: Vestland; locality: Bakkasund; verbatimCoordinates: 60°07'51"N 5°05'31"E; **Event:** eventDate: 03/08/2021; eventRemarks: Nina T. Mikkelsen leg.; **Record Level:** institutionCode: ZMBN; basisOfRecord: PreservedSpecimen
- **Type status:** Other material. **Occurrence:** individualID: ZMBN140294; individualCount: 1; lifeStage: adult; otherCatalogNumbers: BOLD:ADZ1926; associatedSequences: BOLD:NORCH006-21, GenBank: OR526591; occurrenceID: C5272212-FC93-5596-9127-090EB552C728; **Taxon:** scientificNameID: Acanthochitonadiscrepans; **Location:** country: Norway; stateProvince: Vestland; locality: Bakkasund; verbatimCoordinates: 60°07'51"N 5°05'31"E; **Event:** eventDate: 03/08/2021; eventRemarks: Nina T. Mikkelsen leg.; **Record Level:** institutionCode: ZMBN; basisOfRecord: PreservedSpecimen
- **Type status:** Other material. **Occurrence:** individualID: ZMBN140295; individualCount: 1; lifeStage: adult; otherCatalogNumbers: BOLD:ADZ1926; associatedSequences: BOLD:NORCH007-21, GenBank: OR526592; occurrenceID: D519A7ED-0999-53BA-9539-819BC258DA1C; **Taxon:** scientificNameID: Acanthochitonadiscrepans; **Location:** country: Norway; stateProvince: Trondelag; locality: Hopavågen; verbatimCoordinates: 63°35'34"N 9°32'02"E; **Event:** eventDate: 15/10/2020; eventRemarks: Nina T. Mikkelsen leg.; **Record Level:** institutionCode: ZMBN; basisOfRecord: PreservedSpecimen
- **Type status:** Other material. **Occurrence:** individualID: ZMBN46755; individualCount: 1; lifeStage: adult; otherCatalogNumbers: BOLD:ADZ1926; associatedSequences: BOLD:NORCH059-22, GenBank: OR526594; occurrenceID: 6E34B9BD-25B0-55B0-B0D3-3E7A4AE7E5B4; **Taxon:** scientificNameID: Acanthochitonadiscrepans; **Location:** country: Norway; stateProvince: Vestland; locality: Sævrøysund; verbatimCoordinates: 60°48'14"N 4°48'29"E; **Event:** eventDate: 21/06/2021; eventRemarks: Nina T. Mikkelsen leg.; **Record Level:** institutionCode: ZMBN; basisOfRecord: PreservedSpecimen
- **Type status:** Other material. **Occurrence:** individualID: SMF 373034; individualCount: 1; lifeStage: adult; otherCatalogNumbers: BOLD:ADZ1926, NIB_CCM_0471; associatedSequences: BOLD:CCMMO037-21, GenBank: OR526583; occurrenceID: 9F36D135-538C-5B4A-8322-E4135C813101; **Taxon:** scientificNameID: Acanthochitonadiscrepans; **Location:** country: Northern Ireland; county: Down; locality: Strangford Lough; verbatimCoordinates: 54°29'34"N 5°39'03"W; **Event:** eventDate: 25/05/2021; eventRemarks: Christine Morrow leg.; **Record Level:** collectionCode: Malakologie- SMF; basisOfRecord: PreservedSpecimen
- **Type status:** Other material. **Occurrence:** individualID: SMF 373035; individualCount: 1; lifeStage: adult; otherCatalogNumbers: BOLD:ADZ1926, NIB_CCM_0475; associatedSequences: BOLD:CCMMO040-21, GenBank: OR526584; occurrenceID: C8E53288-0BE9-53D7-AD6E-1CCBA45BE000; **Taxon:** scientificNameID: Acanthochitonadiscrepans; **Location:** country: Northern Ireland; county: Down; locality: Strangford Lough; verbatimCoordinates: 54°29'34"N 5°39'03"W; **Event:** eventDate: 25/05/2021; eventRemarks: Christine Morrow leg.; **Record Level:** collectionCode: Malakologie- SMF; basisOfRecord: PreservedSpecimen
- **Type status:** Other material. **Occurrence:** individualID: ZMBN140330; individualCount: 1; lifeStage: adult; otherCatalogNumbers: BOLD:ADZ1926; associatedSequences: BOLD:NORCH108-23, GenBank: OR526586; occurrenceID: 1B7EC7A4-D596-5C8D-8154-A3017FE66F2A; **Taxon:** scientificNameID: Acanthochitonadiscrepans; **Location:** country: Norway; stateProvince: Trondelag; locality: Hopavågen; verbatimCoordinates: 63°35'35"N 9°32"E; **Event:** eventDate: 14/10/2020; eventRemarks: Nina T. Mikkelsen leg.; **Record Level:** institutionCode: ZMBN; basisOfRecord: PreservedSpecimen
- **Type status:** Other material. **Occurrence:** individualID: ZMBN140333; individualCount: 1; lifeStage: adult; otherCatalogNumbers: BOLD:ADZ1926; associatedSequences: BOLD:NORCH110-23, GenBank: OR526588; occurrenceID: E888D7B1-95EF-5C98-9E1E-B109714BB316; **Taxon:** scientificNameID: Acanthochitonadiscrepans; **Location:** country: Norway; stateProvince: Vestland; locality: Espegrend; verbatimCoordinates: 60°16'11"N 5°13'19"E; **Event:** eventDate: 25/05/2021; eventRemarks: Nina T. Mikkelsen leg.; **Record Level:** institutionCode: ZMBN; basisOfRecord: PreservedSpecimen
- **Type status:** Other material. **Occurrence:** individualID: ZMBN140331; individualCount: 1; lifeStage: adult; otherCatalogNumbers: BOLD:ADZ1926; associatedSequences: BOLD:NORCH111-23, GenBank: OR526589; occurrenceID: 342B65CF-9A4E-5005-A996-C5ABB08B0C88; **Taxon:** scientificNameID: Acanthochitonadiscrepans; **Location:** country: Norway; stateProvince: Vestland; locality: Puddefjorden; verbatimCoordinates: 60°22'51"N 5°19'30"E; **Event:** eventDate: 24/08/2021; eventRemarks: Nina T. Mikkelsen leg.; **Record Level:** institutionCode: ZMBN; basisOfRecord: PreservedSpecimen
- **Type status:** Other material. **Occurrence:** individualID: SMF 373025; individualCount: 1; lifeStage: adult; associatedSequences: GenBank: OR145401; occurrenceID: 025E0A78-B0C7-5BCA-BA86-7D6549DA52A4; **Taxon:** scientificNameID: Acanthochitonadiscrepans; **Location:** country: Northern Ireland; county: Down; locality: Strangford Lough; verbatimCoordinates: 54°29'23"N 5°32'15"W; **Event:** eventDate: 09/19/2019; eventRemarks: Julia Sigwart leg.; **Record Level:** collectionCode: Malakologie- SMF; basisOfRecord: PreservedSpecimen
- **Type status:** Other material. **Occurrence:** individualID: ZMBN140296; individualCount: 1; lifeStage: adult; otherCatalogNumbers: BOLD:ADZ1926; associatedSequences: NORCH008-21, GenBank: OR526593; occurrenceID: 4FFE8F85-37DE-54CF-8353-2586579A9A8D; **Taxon:** scientificNameID: Acanthochitonadiscrepans; **Location:** country: Norway; stateProvince: Trondelag; locality: Hopavågen; verbatimCoordinates: 63°35'34"N 9°32'02"E; **Event:** eventDate: 15/10/2020; eventRemarks: Nina T. Mikkelsen leg.; **Record Level:** institutionCode: ZMBN; basisOfRecord: PreservedSpecimen

#### Description

Animal small to medium size (in examined material, BL: 11–22 mm, BW: 5–11 mm, BL/BW ratio: 2.1), outline oval, moderately elevated (elevation ratio 0.41), semi-carinated, side slopes flat to slightly raised, apices not prominent; girdle wide, spiculose (Fig. 1C). Dorsal colouration not uniform, often mottled, variable; tegmentum brownish to dark red, lateropleural and antemucronal areas with blue, dark brown or beige maculation, jugum usually dark brown to black; colour of perinotum beige to pale yellow mottled with olive green or brown; normally 18 sutural tufts (Fig. 1C).

**Figure 5.**
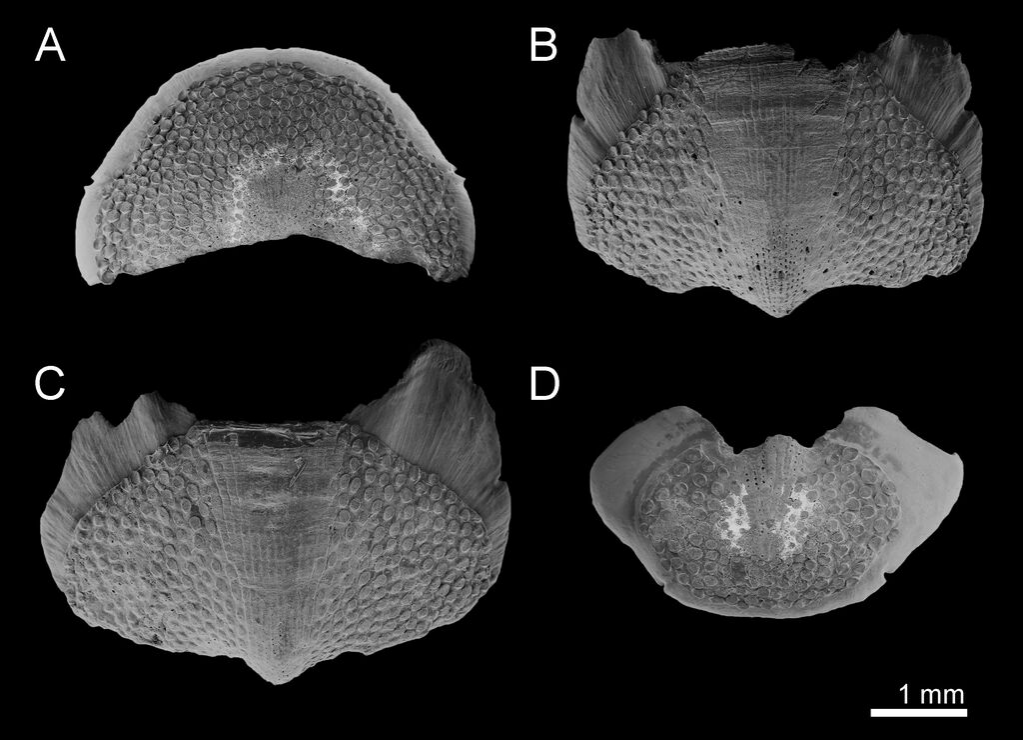
*Acanthochitonadiscrepans* from Northern Ireland (A, D) and Norway (B–C). All in dorsal view. **A** Valve I; **B** Valve V; **C** Valve VII; **D** Valve VIII.

Head valve slightly wider than long, almost semicircular, anterior slope slightly convex, posteriormost margin straight without a notch (Fig. 1C, Fig. 5A). Intermediate valves ellipsoidal, wide, anterior margins slightly rounded at both sides of straight or little concave jugum, side margins rounded, very slightly beaked with hind margin slightly concave at both sides of the apex; smoothly rounded or semi-carinate; jugal areas striated, wide, wedge-shaped, little elevated and hardly separated from the lateropleural areas (Fig. 1C, Fig. 5B, C). Tail valve roughly semicircular with central, moderately-elevated mucro and posterior slope steep and straight (Fig. 5D).

**Figure 6.**
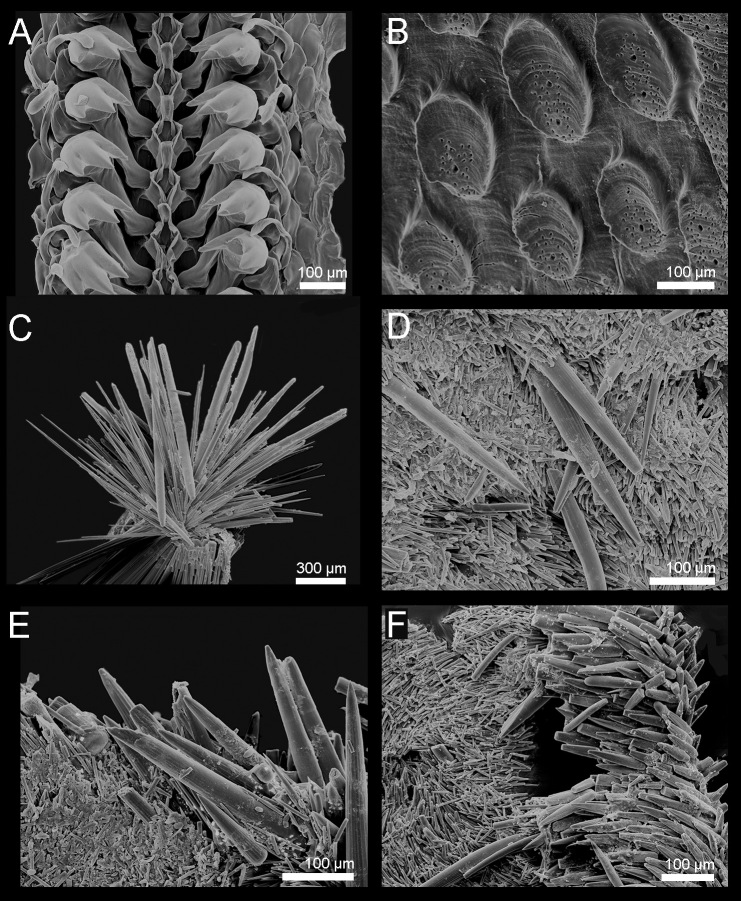
*Acanthochitonadiscrepans* from Northern Ireland (A–E) and Norway (F). **A** Radula; **B** Valve VII, detail of the tegmentum in the lateropleural area; **C** One of the 18 tufts of spicules on the girdle; **D** Dorsal spicules; **E** Dorsal and marginal spicules; **F** Dorsal, marginal and ventral spicules.

Tegmentum uniformly covered with rather densely distributed (density from 24 to 50 granules per 1 mm^2^, mean = 37, n = 8 specimens), oval to elongated granules, arranged in quincunx order, except for the jugal area of intermediate valves; the granules raised, flat topped, to slightly concave (Fig. 1C, Fig. 4B, Fig. 5, Fig. 6B). Proximal granules more elongate and merging into jugum; in lateral areas, granules spaces less than granule-width apart. Each granule with 1–2 macroaesthetes (single macroaesthete located in posterior third; second, if present, located centrally) surrounded by 26–40 very small posteriomedially located microaesthetes (Fig. 4B, Fig. 6B).

Articulamentum well developed, solid, slightly pinkish ventrally beneath tegmentum. Apophyses large, separated at jugum, broadly rectangular to wing-shaped, trapezoidal in tail valve (direct dorsal view can create appearance of triangular outline); insertion plates wide, continuous with apophyses, slits deep, extending proximally into shallow dorsal channels (slit formula: 5/1/2) (Fig. 5).

Girdle wide, fleshy, leathery looking, brighter than tegmentum, deeply encroaching sutural areas. Colour from beige or light yellow to pale brown with brown and olive-green streaks and blotches, in some specimens prominent dark green bands with fuzzy boundaries located near valves between the sutural tufts; sutural tufts with translucent or brownish spicules; base of sutural tufts creamy-yellow or brown (Fig. 1C). Dorsal surface densely covered by minute, thin brownish spicules (up to 68 x 7.5 µm) sculptured with fine riblets, pointed, but often broken (Fig. 6D, F). In some specimens, randomly interspersed longer and thicker spicules, located only in outer half of the girdle identical to spicules of marginal fringe (Fig. 6F). Sutural tufts prominent, with around 15–40 thick, sharply pointed, smooth needles, measuring up to 1000 µm x 60 µm, surrounded by much shorter and thinner, but also smooth bristles (Fig. 6C). Marginal fringe with rounded, solid spicules, ribbed on proximal half, distal half ribbed or smooth, up to 300 x 35 µm. (Fig. 6E–F). Hyponotum densely covered with imbricating flattened, elongated spicules, finely striated near the top with 3-4 ribs, much larger than dorsal spicules, up to 120 x 20 µm (Fig. 6F).

Radula central tooth elongated, without cusp, almost straight at the top, keeled near base; first lateral tooth wing-shaped and wrapping around central; second (major) lateral massive with accessory plate tricuspidate with outer denticle shorter than the others; cusps pointed and triangular in outline (Fig. 6A).

Gills merobranchial, composed of 9–13 ctenidia on each side reaching around half body length.

#### Diagnosis

This species can be distinguished from other *Acanthochitona* species from NE Atlantic on the basis of a set of morphological characters. It differs from *A.crinita* (Pennant, 1777) by the number, size and arrangement of aesthetes on tegmental granules (1–2 macroaesthetes - a single macroaesthete located in posterior third, second, if present, located centrally, surrounded by very small, posteriomedially located 26–40 microaesthetes in *A.discrepans* vs. one macroaesthete subcentral surrounded by larger, posteromedially located 8–16 microaesthetes in *A.crinita*), dorsal spicules (smaller, thinner spicules up to 68 x 7.5 µm in *A.discrepans* vs. longer, thicker spicules up to 100 x 15 µm in *A.crinita*), tuft needles (longer spicules up to 1000 µm x 60 µm, surrounded by a large number of somewhat shorter and much thinner needles in *A.discrepans* vs. longer spicules up to 340 x 20 µm, surrounded by a smaller number of very short and thin needles in *A.crinita*). *Acanthochitonadiscrepans* can be distinguished from *A.fascicularis* (Linnaeus, 1767) by the shape of the intermediate valves (ellipsoidal in *A.discrepans* vs. triangular in *A.fascicularis*), jugal area (hardly raised, not sharply separated from the latero-pleural areas in *A.discrepans* vs. raised, sharply separated from the latero-pleural areas in *A.fascicularis*), shape of dorsal granules (decidedly apart, oval to elongated oval in *A.discrepans* vs small, round, densely packed granules with the incision in the middle in *A.fascicularis*), density of tegmental granules (24–50 granules per 1 mm^2^, mean = 37 in *A.discrepans* vs. 42–70 granules per 1 mm^2^, mean = 52 in *A.fascicularis*), number of microaesthetes (26–40 microaesthetes in *A.discrepans* vs. 0–6 microaesthetes in *A.fascicularis*), number of bristles in the sutural tufts (15–40 in *A.discrepans* vs. 55-120 in *A.fascicularis*). *Acanthochitonadiscrepans* differs from *A.oblonga* Leloup, 1981 by the shape of dorsal granules (rather densely distributed, oval to elongated granules, arranged in quincunx order in *A.discrepans* vs. widely apart and very much elongated in *A.oblonga*), number and arrangement of microaesthetes (26–40 microaesthetes located posteromedially in *A.discrepans* vs. 6–9 microaesthetes located mainly in the central area in *A.oblonga*). It can be distinguished from *A.pilosa* Schmidt-Petersen, Schwabe et Haszprunar, 2015 on the basis of the shape of the IV valve (very slightly-beaked valve with hind margin slightly concave at both sides of the apex in *A.discrepans* vs. valve with triangular posterior margin with no apex in *A.pilosa*), number of microaesthetes on the tegmental granules (26–40 microaesthetes in *A.discrepans* vs. around 14 microaesthetes in *A.pilosa*), large dorsal spicules (only sometimes present and very sparse large dorsal spicules in *A.discrepans* vs. always present, dense and straight spicules in *A.pilosa*).

#### Distribution

This species seems to have a more northern distribution than *Acanthochitonacrinita*. The range of species is from Norway (molecular data and SEM photos from this study), through the north coast of Ireland (molecular data and SEM photos from this study) to the south of Wales (*locus typicus*). The literature records from more southern regions must be treated with caution as they probably represent other misidentified *Acanthochitona* species.

#### Taxon discussion

The original *locus typicus* of *Acanthochitonadiscrepans* (Brown, 1827) is in Tenby, Pembroke in Wales. Kaas (1985) chose the lectotype from the syntype series (Lyons collection, Tenby Museum). In our study, we re-describe this species, based on the specimens from the Strangford Lough from the Northern Ireland (ca. 320 km north from the *locus typicus*). However, our specimens in all aspects correspond to the description provided by Kaas (1985); Kaas also examined the specimens from Strangford Lough, confirmed that they are *A.discrepans* and stated that this species is fairly common there.

### Acanthochitona fascicularis

(Linnaeus, 1767)

A5E322E1-6788-58D9-90B4-481007AF9737

https://www.marinespecies.org/aphia.php?p=taxdetails&id=138677

Chiton fascicularis : Linnaeus, 1767 - Linnaeus 1767: 1106; Poli, 1791 - Poli 1791: 10, pl. 4 fig. 3; Reeve, 1847 - Reeve 1827: pl. 10 sp. & fig. 53.
non *C.fascicularis*: Sowerby, 1840a - Sowerby II 1840a: figs. 87, 87a, et mult. auct.
Acanthochites communis : Risso, 1826 - Risso 1826: 269.
Chiton fascicularis var. major : Philippi, 1836 - Philippi 1836: 108, pl. 7 fig. 2a, b.
Chiton crinitus : Sowerby, 1840: figs. 88-93; 1840a: 2.
non *C.crinitus*: Pennant, 1777 - Pennant 1777.
Chiton discrepans : Sowerby, 1840a: 2 (in synonymy of *C.crinitus*; Sowerby, non Pennant, 1777); Forbes & Hanley, 1849 - Forbes and Hanley 1849: 396, pl. 58 fig. 4; Jeffreys, 1859 - Jeffreys 1859: 106, pl. 3 fig. 10, Jeffreys, 1869 - Jeffreys 1869: 198, pl. 5. fig. 4, et mult, auct.
non *C.discrepans*: Brown, 1827 - Brown 1827.
Acanthochites carinatus : H. Adams & Angas, 1864 Adams and Angas 1864.
non *Acanthochitescarinatus*: Risso, 1826 - Risso 1826.
Chiton fascicularis var. rubra : Issel, 1870 - Issel 1870: 4.
Acanthochites discrepans var. minorflava : di Monterosato, 1878a - di Monterosato 1878: 78.
Acanthochites hamatus : de Rochebrune, 1882 - de Rochebrune 1882: 191; Thiele, 1909 - Thiele 1909: 43.
Anisochiton discrepans vars *elongata*, *marmorata*, *nigrolineata*: Dautzenberg, 1893 - Dautzenberg 1893: 25.
Acanthochites discrepans var. albina : Dautzenberg & Durouchoux, 1900 - Dautzenberg and Durouchoux 1900: 15.
Anisochiton (Acanthochites) discrepans var. viridis : Pallary, 1902 - Pallary 1902: 28.
Acanthochites discrepans var. violaceolimbata : Dautzenberg & Durouchoux, 1906 - Dautzenberg and Durouchoux 1906: 15.
*Acanthochiton(a) communis*: Winckworth, 1926 - Winckworth 1926: 15, et mult. auct.
Acanthochiton heterochaetus : Bergenhayn, 1931 - Bergenhayn 1931: 20, pl. 1 figs. 38-42, pl. 3 figs 67-74.
Acanthochiton discrepans var. angustivalvus : Bergenhayn, 1931 - Bergenhayn 1931: 20.
Acanthochiton communis f. barashi : Leloup, 1969 - Leloup 1969: 1, figs. 1, 2D, 3D, G, 4B.
Acanthochitona bonairensis : Kaas, 1972 - Kaas 1972: 44, text figs 72-73, pl. 3 figs. 1, 2; Watters, 1981 - Watters 1981: 173 (in synonymy of *A.communis*).
Acanthochitona fascicularis : Kaas, 1985 - Kaas 1985: 585, figs. 1-6.
**Type material.** Neotype: MNHN-IM-2000-5923, Muséum National d’Histoire Naturelle, Paris, France. Designated by Kaas (1985) (material not seen).
**Type locality.** Algeria, coast of Barbary (Linnaeus 1767); Algeria, Oran (Kaas 1985).

#### Materials

- **Type status:** Other material. **Occurrence:** individualID: SMF 373027; individualCount: 1; lifeStage: adult; otherCatalogNumbers: BOLD:AAJ3296, NIB_CCM_0522; associatedSequences: BOLD:CCMMO043-21, GenBank: OR526595; occurrenceID: 55E89EFE-E8B6-5D86-9019-A160AFE8A3EF; **Taxon:** scientificNameID: Acanthochitonafascicularis; **Location:** country: Ireland; county: Galway; locality: Bealadangan; verbatimCoordinates: 53°18'43"N 9°37'26"W; **Event:** eventDate: 21/09/2021; eventRemarks: Christine Morrow leg.; **Record Level:** collectionCode: Malakologie- SMF; basisOfRecord: PreservedSpecimen
- **Type status:** Other material. **Occurrence:** individualID: SMF 373030; individualCount: 1; lifeStage: adult; otherCatalogNumbers: BOLD:AAJ3296, NIB_CCM_0537; associatedSequences: BOLD:CCMMO046-21, GenBank: OR526596; occurrenceID: 6110752C-8064-5DE9-97C2-8AEC5CDCECFD; **Taxon:** scientificNameID: Acanthochitonafascicularis; **Location:** country: Ireland; county: Galway; locality: Aillwee; verbatimCoordinates: 53°21'21"N 9°38'45"W; **Event:** eventDate: 24/09/2021; eventRemarks: Christine Morrow leg.; **Record Level:** collectionCode: Malakologie- SMF; basisOfRecord: PreservedSpecimen
- **Type status:** Other material. **Occurrence:** individualID: SMF 373028; individualCount: 1; lifeStage: adult; otherCatalogNumbers: BOLD:AAJ3296, NIB_CCM_0523; associatedSequences: BOLD:CCMMO044-21, GenBank: OR526597; occurrenceID: 474B14EF-848E-5AB3-9408-AE88C534A0F1; **Taxon:** scientificNameID: Acanthochitonafascicularis; **Location:** country: Ireland; county: Galway; locality: Bealadangan; verbatimCoordinates: 53°18'43"N 9°37'26"W; **Event:** eventDate: 20/09/2021; eventRemarks: Christine Morrow leg.; **Record Level:** collectionCode: Malakologie- SMF; basisOfRecord: PreservedSpecimen
- **Type status:** Other material. **Occurrence:** individualID: 360506-4; individualCount: 1; lifeStage: adult; associatedSequences: GenBank: OR145409; occurrenceID: 459DF758-0C45-5ACA-8467-6F6403CEE765; **Taxon:** scientificNameID: Acanthochitonafascicularis; **Location:** country: Portugal; stateProvince: Azores; county: Galway; locality: Ponta Delgada: Rosto do Cão; verbatimCoordinates: 37°44.567'N 25°38.167'W; **Event:** eventDate: 29/07/2013; eventRemarks: Julia Sigwart, Laura Sumner-Rooney & Nicholas Carey leg.; **Record Level:** collectionCode: Malakologie- SMF; basisOfRecord: PreservedSpecimen
- **Type status:** Other material. **Occurrence:** individualID: SMF 373029; individualCount: 1; lifeStage: adult; otherCatalogNumbers: BOLD:AAJ3296, NIB_CCM_0524; associatedSequences: BOLD:CCMMO045-21, GenBank: OR526598; occurrenceID: F1DC0BB9-0B94-5DEE-A0A8-766B86D322C2; **Taxon:** scientificNameID: Acanthochitonafascicularis; **Location:** country: Ireland; county: Galway; locality: Bealadangan; verbatimCoordinates: 53°18'43"N, 9°37'26"W; **Event:** eventDate: 20/09/2021; eventRemarks: Christine Morrow leg.; **Record Level:** collectionCode: Malakologie- SMF; basisOfRecord: PreservedSpecimen

#### Description

(See Jones and Baxter (1987), Dell’Angelo and Smriglio (2001), Bonfitto et al. (2011), Schmidt-Petersen et al. (2015) for additional descriptions that pertain to *Acanthochitonafascicularis*). Small to large size (in examined material, BL: 9–30 mm, BW: 5–18 mm, BL/BW ratio: 1.7), outline oval, flat to moderately elevated, semi-carinated, slightly protruding apices; girdle very wide and spiculose. Dorsal colouration variable; usually green and brown variously combined; some specimens can be uniform and brightly coloured, yellow or orange (Fig. 1D–E).

**Figure 7.**
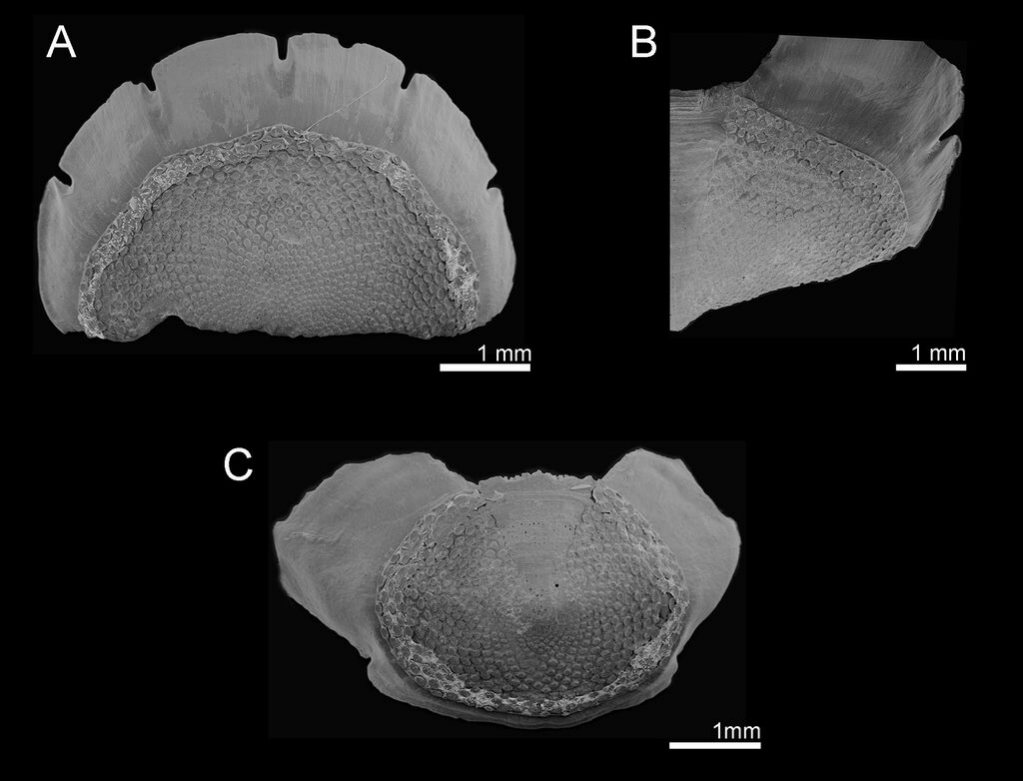
*Acanthochitonafascicularis* from Ireland. All in dorsal view. **A** Valve I; **B** Valve V; **C** Valve VIII.

Head valve much wider than long, semicircular, with five barely-raised rays, posterior margin almost straight, no notch and apex (Fig. 7A). Intermediate valves from triangular to trapezoid, apex slightly protruding; raised, clearly separated from lateropleural areas, wedge-shaped and striated jugum (Fig. 1D–E, Fig. 7B). Tail valve almost circular with central elevated mucro (Fig. 1D, Fig. 6C).

**Figure 8.**
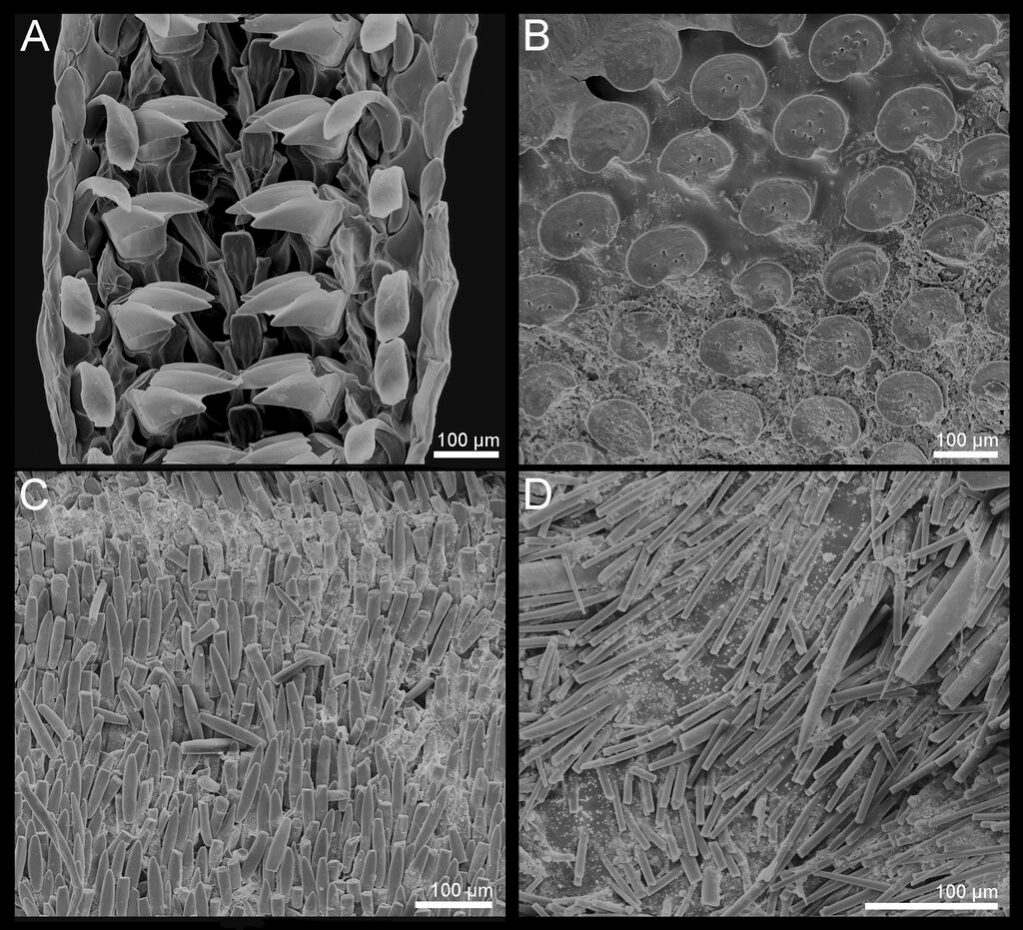
*Acanthochitonafascicularis* from Ireland. **A** Radula; **B** Valve V, detail of the tegmentum in the lateropleural area; **C** Ventral spicules; **D** Dorsal spicules.

Tegmentum uniformly and thickly covered (density from 42 to 70 granules per 1 mm^2^, mean = 52, n = 7 specimens) by small, round granules arranged in arched lines on the valves; each granule with 0–6 aesthetes randomly distributed, without a clear separation between macro- and microaesthetes; some granules with an incision in the middle of the granule posterior margin (Fig. 1D, Fig. 4C, Fig. 7, Fig. 8B).

Articulamentum well-developed; apophyses wide, rectangular, separated at jugum, rounded at anterolateral margins; insertion plates wide, continuous with apophyses, slits deep, extending dorsally into shallow channels (slit formula: 5/1/2) (Fig. 7).

Girdle very wide and fleshy, deeply encroaching sutural areas. Colours ranging from creamy-yellow to olive-green and brown, with different degrees of blotches in which white, green and brown variously combined; sometimes yellow or orange (Fig. 1D–E). Dorsal perinotum densely covered with two kinds of spicules: shorter, slender, pointed, but usually broken spicules and longer and thicker spicules interspersed amongst them (Fig. 8D). Sutural tufts with 55–120 long and slim bristles, surrounded by shorter, thinner, also smooth needles. Hyponotum densely covered with lanceolate, flattened spicules, finely striated near the top, of size up to 100 x 18 µm (Fig. 8C).

Central radula teeth elongated, almost straight at the top and keeled near base; first lateral tooth wing-shaped and smaller than the central; second (major) lateral elongated with accessory plate tricuspidate with central denticle longer than the others; cusps pointed and triangular in outline (Fig. 8A).

Gills merobranchial, 15-17 ctenidia on each side.

#### Diagnosis

*Acanthochtionafascicularis*, can be easily distinguished from other NE Atlantic relatives by the triangular shape of valves, raised and clearly separated jugal area, small and round, densely-packed tegmental granules and small number of aesthetes (0–6) without clear separation between micro- and macroaesthetes. For comparison, see the Remarks section for *A.crinita* and *A.discrepans*.

#### Distribution

This species is widely distributed in the North-East Atlantic, with a range from the west coast of Ireland (molecular data and SEM photos from this study), south to the Azores (molecular data and SEM photos from this study) and the Mediterranean Sea (literature records and GenBank sequences).

## Analysis

**Phylogenetics results**

**Figure 9.**
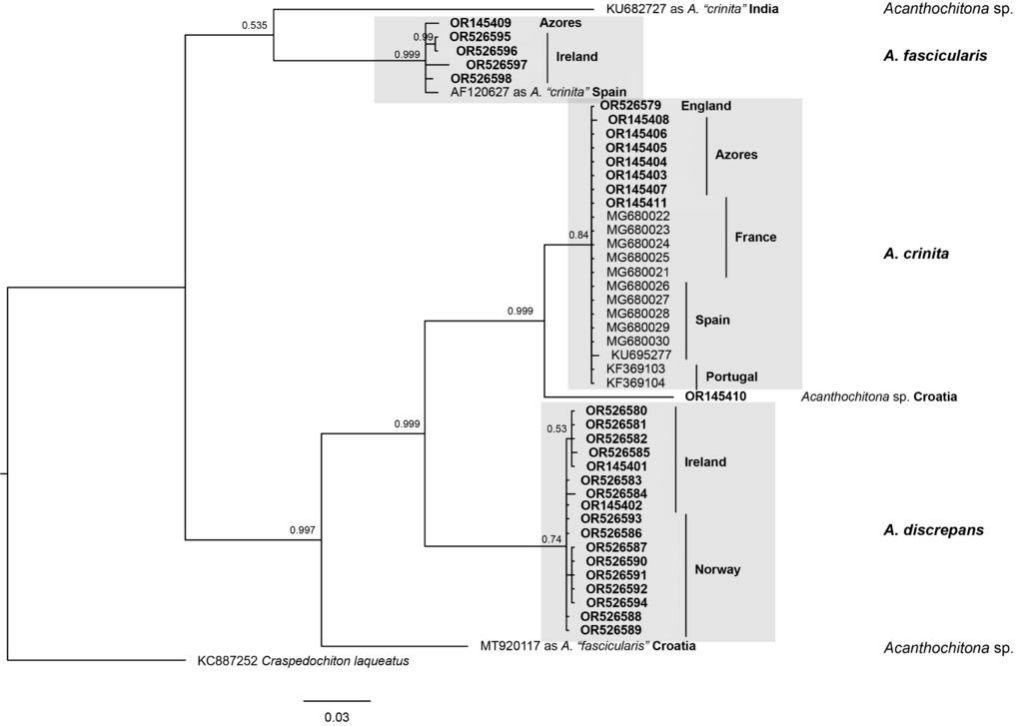
Bayesian phylogenetic reconstruction of the North-East Atlantic *Acanthochitona*, based on newly-sequenced specimens (in bold) and sequences downloaded from GenBank. Values at nodes are BI posterior probability supports.

The consensus tree obtained through Bayesian Inference is shown in Fig. 9. The tree shows three distinct and well-supported clades: *Acanthochitonacrinita* and *A.discrepans* as sister species, together forming a clade which shows a sister relationship to *Acanthochitonafascicularis*. The *A.crinita* clade includes also one specimen from Croatia (OR145410) which may be a separate undescribed species. Three sequences from GenBank represent misidentified specimens: KU682727 from India labelled as "*A.crinita*" is a sister lineage to *A.fascicularis* s.s. and of unknown affinity; AF120627 from Spain labelled as "*A.crinita*" in fact represents *A.fascicularis* s.s.; MT929117 from Croatia labelled as "*A.fascicularis*" is a sister species to the clade (*A.crinita* + *A.discrepans*) and represents an unknown species.

A total of 32 new COI sequences and 17 sequences downloaded from GenBank of a length of 618 bp were used for the phylogenetic analysis. No stop codons or gaps in the alignment were present. Uncorrected pairwise distances in COI sequences between the three analysed species were as follows: 14.7–15.7% between *Acanthochitonafascicularis* and *A.crinita*; 13.6–14.7% between *A.fascicularis* and *A.discrepans*; 10.8–11.3% between *A.crinita* and *A.discrepans* (uncorrected pairwise distances in COI between all sequences included in the phylogenetic analysis can be found in Suppl. material 3).

## Discussion

*Acanthochitonacrinita*, *A.discrepans* and *A.fascicularis* were confused for a very long time, but do constitute three distinct species. They can be separated, based on the morphology and differ also in their geographic distribution. *Acanthochitonafascicularis* has a very wide distribution, it inhabits seas from the west coast of Ireland to the Mediterranean Sea, *A.crinita* occurs from Scotland to the west coast of Portugal and, most likely, in the Mediterranean Sea. *A.discrepans* represents a more northern species than *A.crinita*, which has a range from northern Norway, along the north Irish coasts to the south of Wales; the ranges of *A.crinita* and *A.discrepans* probably overlap in Scotland and Ireland.

Taxonomy of *Acanthochitona* has been mostly based on the shape and distribution of tegmental granules combined with the girdle ornamentation; however, these characters in isolation tend to be variable and are not diagnostic (e.g. Leloup (1941), Leloup (1968), Dell’Angelo and Smriglio (2001)). The most reliable approach is combined observations of the shape and distribution of tegmental granules with characters of their structure, especially the size, number and arrangement of micro- and macroaesthetes (Leloup 1941, Bonfitto et al. 2011, Schmidt-Petersen et al. 2015). Little is known about the quantitative intra- and interspecific variability of these structures. For effective species discrimination, they should be accompanied by molecular data whenever possible.

The three NE Atlantic *Acanthochitona* require careful observation in order to be separated with confidence. *Acanthochitonafascicularis* is the most distinct species, with its large size, very broad girdle and small, densely-arranged granules. *Acanthochitonacrinita* and *A.discrepans* are superficially similar and confident separation should be made, based on SEM photos or DNA barcodes. Assessment of detailed granule structure is only practicable with SEM. Girdle characters, although less reliably diagnostic, can be useful in field identification. The girdle of *A.fascicularis* is much broader than in the two other species and densely covered with very short thin spicules, but with spines of different lengths interspersed; *A.crinita* usually has the girdle covered with two kind of spines: short, thick spines and much longer, thicker and curved spines between them; *A.discrepans* has the cover of very short, densely-arranged spicules which give the girdle a more leathery look.

*Acanthochitonacrinita*, *A.discrepans* and *A.fascicularis* are difficult to tell apart and past records must be treated with caution; however, with our new data, there is an emergent pattern in their distributions that helps to clarify how these species are separated. *Acanthochitonafascicularis* can be found from the west coasts of Ireland to the Azores and the Mediterranean Sea (Kaas 1985, Bonfitto et al. 2011, Ávila and Sigwart 2013, Mitov 2015). The very broad range inferred from literature is beyond what is biogeographically plausible and records from remote localities, such as the Falkland Islands (Leloup 1941) should be considered misidentifications. *Acanthochitonadiscrepans* appears to be a northern species, distributed along the coast of Norway, in the north of Ireland and to the south of Wales. Literature records of *A.discrepans* from the Azores (MacAndrew 1856) and the Mediterranean (Dautzenberg and Durouchoux 1900) are most likely misidentifications. The geographic ranges of these two species are now relatively clear, but the old records, especially from remote localities, cannot be considered reliable.

*Acanthochitonacrinita* has always been challenging for taxonomists. It remains difficult to assess its geographic ranges because it is highly variable and apparently genuinely occupies a very broad range, overlapping with other *Acanthochitona* species. The confirmed range extends from the north of Scotland, along the coast of England, France, Spain and Portugal to the Azores. There are dozens of records of this species from North-East Atlantic: Iceland, Norway, Scotland, Ireland, Mediterranean Sea, Black Sea, Cape Verde Archipelago and Sao Tome and Principe Islands, but most of them are questionable (e.g. Kaas (1985), Dell’Angelo and Smriglio (2001), Peñas et al. (2006), Bonfitto et al. (2011), Dell’Angelo et al. (2014), Granpoder et al. (2023)). All the specimens of *A.crinita* from Norway that we examined turned out to be *A.discrepans*. It is unlikely that *A.crinita* occurs further north than Scotland (*contra* Kaas (1985)) and records from Iceland are especially dubious. We consider as valid the records from Italy (Bonfitto et al. 2011) and from the Black Sea (Mitov 2015), based on the detailed illustration of tegmental granules; however, further molecular research in the Mediterranean Sea and on the west coast of Africa is needed.

The very wide apparent range of *Acanthochitonacrinita* (from Scotland to the west coast of Africa as far south as São Tomé and Príncipe Islands), its well-known high polymorphism, different descriptions by different authors and confused nomenclature may be a sign of the existence of a species complex in North-East Atlantic. In the past, *a priori* assumption of its high variability has led to assigning all morphotypes that did not fit the description of *A.fascicularis* to *A.crinita* and, hence, the longstanding confusion about the validity of *A.discrepans*. Some authors provided records without further description (e.g. Peñas et al. (2006), Dell’Angelo et al. (2014)) so it is difficult to speculate about the proper affinities and some papers illustrated distinct morphotypes assigned to *A.crinita* which were later recognised as a separate species (Dell’Angelo and Smriglio 2001, Schmidt-Petersen et al. 2015). We observed high polymorphism in *A.crinita* within and amongst the populations. *Acanthochitonacrinita* may constitute a “waste basket” of species still waiting to be described or it may represent one lineage with high morphological and molecular variability. The relatively recent discovery of two new species (*A.oblonga* from Malta and *A.pilosa* from the south coast of France) hint at the likely presence of a species complex in the Mediterranean Sea and west Africa.

The three *Acanthochitona* species considered herein are very variably coloured, but we noticed that specimens of multiple *Acanthochitona* species from Ireland, N. Ireland and England are usually more brownish or greenish, whereas those from Norway and the Azores are more diversely and vividly coloured. This could reflect the local environment in context of the substratum. Richness and variability of the background colour has been associated with higher colour polymorphism in other chiton populations; random colours of chitons on a complex and mixed colour background is thought to help avoid predation by visual predators (Rodrigues and Absalão 2005, Mendonça et al. 2015). Highly-coloured habitats or higher water clarity in Norway and the Azores may result in local chiton populations being more colourful. There is also apparently a size difference between the Azorean and Irish specimens of *Acanthochitonafascicularis*: Azorean specimens of *A.fascicularis* are relatively small, whereas Irish specimens reach large body size (max BL: 15 mm vs. max BL: 30 mm, respectively).

Northern Ireland is located at a junction between southern warm waters and northern cold waters, which results in very high marine biodiversity in this region. Unfortunately, ongoing climate changes have led to shifting ranges in many marine animals (e.g. Poloczanska et al. 2013, Poloczanska et al. (2016), Poloczanska et al. (2013) Hastings et al. (2020)). *Acanthochitonadiscrepans* seems to be one of these northern species (Norway, Northern Ireland, Wales) with the southern boundary in southern Wales and a similar distribution pattern to other northern species, such as the chiton *Boreochitonruber* (Linnaeus, 1767) and the sea star *Leptasteriasmuelleri* (M. Sars, 1846) (Van Belle and Kaas 1998, Southward and Campbell 2006). With the increase in seawater temperature, more Arctic species will probably move northwards following the general trend of marine taxa (Poloczanska et al. 2013). In the future, *A.discrepans* may shift its range towards more northern waters, so it is especially important to monitor this species in the southern part of its range as it can be an indicator of climate change.

## Supplementary Material

XML Treatment for Acanthochitona crinita

XML Treatment for Acanthochitona discrepans

XML Treatment for Acanthochitona fascicularis

B94BCB5A-DB96-52FC-89BD-CD2CE3AA3A9C 10.3897/BDJ.11.e109554.suppl1 Supplementary material 1 Accession numbers for the sequences downloaded from GenBank. Data type accession numbers File: oo_900103.xlsx https://binary.pensoft.net/file/900103 Katarzyna Vončina

6B99EC23-7160-5A38-832E-33FED25572F3 10.3897/BDJ.11.e109554.suppl2 Supplementary material 2 The alignment of the new sequences from this study and sequences downloaded from GenBank. Data type DNA sequences File: oo_900104.fas https://binary.pensoft.net/file/900104 Katarzyna Vončina

A9FC0619-F737-5F43-959C-C0C826866641 10.3897/BDJ.11.e109554.suppl3 Supplementary material 3 Uncorrected pairwise distances of the new sequences from this study and sequences downloaded from GenBank. Data type genetic distances File: oo_900105.xls https://binary.pensoft.net/file/900105 Katarzyna Vončina
